# SMN Is Physiologically Downregulated at Wild-Type Motor Nerve Terminals but Aggregates Together with Neurofilaments in SMA Mouse Models

**DOI:** 10.3390/biom12101524

**Published:** 2022-10-20

**Authors:** Julio Franco-Espin, Alaó Gatius, José Ángel Armengol, Saravanan Arumugam, Mehri Moradi, Michael Sendtner, Jordi Calderó, Lucia Tabares

**Affiliations:** 1 Department of Medical Physiology and Biophysics, School of Medicine, University of Seville, 41009 Seville, Spain; 2 Department of Experimental Medicine, School of Medicine, University of Lleida, Institut de Recerca Biomèdica de Lleida (IRBLleida), 25198 Lleida, Spain; 3 Department of Physiology, Anatomy and Cell Biology, University Pablo de Olavide, 41013 Seville, Spain; 4 Institute of Clinical Neurobiology, University Hospital Wuerzburg, 97078 Wuerzburg, Germany

**Keywords:** spinal muscular atrophy, motor neuron degeneration, SMN granules, neuromuscular junction, *β-actin* mRNA, MAP1B, neurofilaments

## Abstract

Survival motor neuron (SMN) is an essential and ubiquitously expressed protein that participates in several aspects of RNA metabolism. SMN deficiency causes a devastating motor neuron disease called spinal muscular atrophy (SMA). SMN forms the core of a protein complex localized at the cytoplasm and nuclear gems and that catalyzes spliceosomal snRNP particle synthesis. In cultured motor neurons, SMN is also present in dendrites and axons, and forms part of the ribonucleoprotein transport granules implicated in mRNA trafficking and local translation. Nevertheless, the distribution, regulation, and role of SMN at the axons and presynaptic motor terminals in vivo are still unclear. By using conventional confocal microscopy and STED super-resolution nanoscopy, we found that SMN appears in the form of granules distributed along motor axons at nerve terminals. Our fluorescence in situ hybridization and electron microscopy studies also confirmed the presence of *β-actin* mRNA, ribosomes, and polysomes in the presynaptic motor terminal, key elements of the protein synthesis machinery involved in local translation in this compartment. SMN granules co-localize with the microtubule-associated protein 1B (MAP1B) and neurofilaments, suggesting that the cytoskeleton participates in transporting and positioning the granules. We also found that, while SMN granules are physiologically downregulated at the presynaptic element during the period of postnatal maturation in wild-type (non-transgenic) mice, they accumulate in areas of neurofilament aggregation in SMA mice, suggesting that the high expression of SMN at the NMJ, together with the cytoskeletal defects, contribute to impairing the bi-directional traffic of proteins and organelles between the axon and the presynaptic terminal.

## 1. Introduction

Survival motor neuron (SMN) is a 38-kDa, ubiquitously expressed and multifunctional protein involved in distinct aspects of RNA homeostasis, ranging from transcription to translation [1]. SMN is encoded by the *SMN1* gene mapped to chromosome 5q13 [2]. In humans, an inverted duplication in the *SMN1* region results in a second centromeric gene called *SMN2* [3]. The main difference between these genes is a C-to-T transition located in exon 7 of *SMN2*. As a result, most *SMN2* transcripts lack exon 7, leading to the production of low levels (~10%) of full-length (FL) SMN protein and ~90% of a truncated and unstable SMNΔ7 protein isoform [4,5]. The deficiency or loss of function of SMN resulting from the homozygous mutation of *SMN1* causes spinal muscular atrophy (SMA), the most common form of motor neuron disease in children, characterized by a severe and progressive neuromuscular pathology [2].

The best-known cellular function of SMN is to act as part of a complex that facilitates the assembly of small U-type ribonucleoproteins (snRNPs) in the cytosol, before they are imported into the nucleus. SMN and gemins form heteromultimeric complexes that incorporate the Sm protein ring into the snRNA [6]. snRNPs are critical for mRNA maturation, by recognizing splicing sites and removing pre-mRNA introns [7]. In addition, SMN has also been identified in the dendrites, axons, and growth cones of cultured motor neurons [8]. Axonal SMN complexes essentially lack Sm proteins [9], indicating that SMN has a different function in the peripheral subcompartments, as supported by the finding that several mutations in SMN not affecting snRNP assembly failed to rescue motor axon defects in SMA [10]. Growing evidence indicates that SMN is a housekeeping protein in mRNA translocation to peripheral subcellular compartments forming part of the macromolecular messenger ribonucleoprotein (mRNP) transport granule family. SMN granules contain several mRNPs, such as hnRNP R, HuD, and IMP1, as well as transcripts, such as *β-actin*, *cpg15*, and *GAP43* mRNAs, which are essential for the growth, differentiation, and maturation of nerve terminals [11,12,13,14,15,16,17,18,19]. SMN-deficiency alters the axonal localization of polyadenylated mRNAs and induces a generalized alteration of the axonal transcriptomic profile in cultured motor neurons [18,20]. Though considerable evidence exists for SMN participation in local translation in vitro [12,15,17,18,21,22,23,24,25,26], it is still unknown whether the essential components of the local translation machinery are present in postnatal motor nerve terminals in vivo, both in control and SMA mice.

Thus, we studied the spatiotemporal organization of SMN in ex vivo nerve terminals in wild-type (non-transgenic) mice and two mouse models of SMA. We hypothesized that peripheral SMN is a key element for the development and maturation of motor nerve terminals. We show that mouse endogenous (Smn) and human transgenic (SMN) proteins form granules distributed along motor axons and are arranged orderly within the presynaptic nerve terminal. In wild-type mice, the density of axonal and preterminal granules decreases after the first week of life, becoming hard to detect in adulthood. By contrast, the heterologous expression of SMN at these compartments in transgenic control and SMA mice remains relatively high after this period. Likewise, our results reveal a potential physiological association between SMN granules and the cytoskeleton, especially with neurofilaments (NFs). However, SMN and NFs form aggregates in SMA mice in axons and nerve terminals, possibly contributing to neuromuscular junction (NMJ) collapse.

## 2. Materials and Methods

### 2.1. Mouse Models

Three murine models with FVB/N background were used: wild-type mice and two mouse models of SMA, the SMN∆7 mouse (FVB.SMNΔ7;SMN2;Smn-, Jackson labs strain no. 005025, [27]), and the so-called Taiwanese mouse (FVB.Cg-Tg(SMN2)2Hung Smn1tm1Hung/J, strain no. 005058 [28]). For each SMA model, experimental mice were grouped into either transgenic control or SMA. Control SMN∆7 mice (*Smn^+/+^; SMN2^+/+^; SMNΔ7^+/+^*) were homozygous for the murine *Smn* gene, and control Taiwanese mice (*Smn^+/−^; SMN2^+/0^*) were heterozygous for the murine gene. SMA mice were: *Smn^−/−^; SMN2^+/+^; SMNΔ7^+/+^* and *Smn^−/−^; SMN2^+/0^*, for the SMN∆7 and Taiwanese lines, respectively. Mice were kept under standard conditions (12:12 light hours:dark and ad libitum feeding). The mice’s genotype was identified using PCR. All experiments were carried out following the guidelines of the European Council Directive for the Care of Laboratory Animals and the animal care and ethics committee of the University of Seville (10/11/2020/128), the University of Lleida (CEEA 04/02-20), and the University of Würzburg (mouse procedures were conducted in accordance with the regulations on animal protection of the German federal law and of the Association for Assessment and Accreditation of Laboratory Animal care, in agreement with the local authorities).

### 2.2. Neuromuscular Preparations

Mice were sacrificed by decapitation and exsanguinated. The transversus abdominis (TVA) muscle was dissected with its nerve branches intact and pinned to the bottom of a 2-mL chamber over a bed of cured silicone rubber. Preparations were continuously perfused with a solution containing (in mM): NaCl 135, KCl 4, CaCl_2_ 2, MgCl_2_ 1, NaHCO_3_ 15, NaH_2_PO_4_ 0.33, and glucose 10. The solution was continuously gassed with 95% O_2_ and 5% CO_2_.

### 2.3. Neuromuscular in Situ Hybridization

The fluorescent in situ hybridization (FISH) technique used in this work consisted of the hybridization of the RNA of interest with a specific oligonucleotide probe of the LNA type (blocked nucleic acid), conjugated with the digoxigenin (DIG) peptide. All steps were carried out at room temperature, if not mentioned otherwise, and all manipulations of the reagents and preparations were carried out in an RNase-free environment. Neuromuscular preparations of the TVA muscle from wild-type mice at P6 were fixed with paraformaldehyde lysine phosphate (PLP) buffer, containing 4% paraformaldehyde (PFA), 75 mM lysine, and 0.01 M sodium meta- periodate, pH 7.4, for 90 min at 4 °C, washed three times with RNase-free PBS, and permeabilized with 1% (*v*/*v*) Triton X-100 in PBS. Proteinase K was added at 2 mg/mL dilution to unmask mRNAs from bound proteins, incubated for 15 min at 37 °C, refixed with PLP for 20 min, and washed with 0.1% (*v*/*v*) Triton X-100 in PBS. For prehybridization, hybridization buffer containing 50% formamide, 0.1% Triton X-100, 9.2 mM citric acid, 50 µg/mL heparin, and 0.5 mg/mL E. coli tRNA in 5× SSC was added to the preparation and incubated 3 h at 40 °C. Forty nM DIG–labeled oligonucleotide probe (LNA probes/Exiqon: *β-actin*/5DigN/ACGCGACCATCCTCCTCTTA/3Dig_N/) was diluted in the hybridization buffer and applied at 40 °C overnight. Afterward, preparations were transferred into 4 sequential incubations with increasing concentration of SSC for 15 min each to 2× SSC/0.1% Triton X-100, followed by incubation with 0.2× SSC/0.1% Triton X-100 for 1 h. The same process was followed to transfer preparations to PBS/0.1% Triton X-100. Finally, for detecting the DIG peptide (mouse monoclonal, Abcam, ab420), Tau (rabbit polyclonal, Sigma, T6402), NF-H (chicken polyclonal, Millipore, ab5539), or AChR (BTX-Cy3); the protocol used was as described below. Samples were mounted with Aqua Poly/Mount mounting medium (Polysciences).

### 2.4. Electron Microscopy

Electron microscopy (EM) was performed as previously reported [29]. Briefly, the tibialis anterior (TA) muscle was dissected and fixed for 1 h at 4 °C in 1% glutaraldehyde and 1% PFA in 0.1 M phosphate buffer (pH 7.4). After that, slabs of the muscle were post-fixed in 1% OsO_4_, stained in the block with 1% uranyl acetate in 70% ethanol, dehydrated, and embedded in Epon 812 (Electron Microscopy Sciences, Fort Washington, PA, USA). Toluidine blue-stained 1-µm semithin sections were cut, till obtaining neuromuscular junctions; then, 60–70-nm ultrathin sections were cut from these areas, collected on copper 300 mesh grids, and examined in a Zeiss Libra 120 EM microscope (CITIUS). Acetate or lead citrate counterstaining was not used, to avoid undesirable precipitates.

### 2.5. Western Blot

Spinal cords and TVA and obliquus abdominis (OA) muscles from wild-type, transgenic control (P10 and P35), and SMA SMN∆7 (P10) mice were used for Western blot analysis. Frozen samples were fragmented and homogenized using an electric homogenizer with ice-cold RIPA lysis buffer (50 mM Tris-HCl [pH 7.4], 150 mM NaCl, 1 mM EDTA, 1% NP-40, 1% Na-deoxycholate, 0.1% SDS) supplemented with protease inhibitor (Sigma-Aldrich, Saint Louis, MO, USA) and PhosSTOP (Roche, Laval, QC, Canada). The homogenized samples were centrifuged at 12,000× *g* rpm for 20 min at 4 °C. The protein concentrations of supernatants were determined by BIO-RAD Micro DC protein assay (BIO-RAD, Laboratories Inc., Hercules, CA, USA). Loading buffer 6 × SS (0.35M Tris-HCl, 10.28% (*w*/*v*) SDS, 36% (*v*/*v*) glycerol, 0.012% (*w*/*v*) bromophenol blue [pH 6.8]) containing 5% β-mercaptoethanol (Sigma-Aldrich), and 20 µg of protein were loaded into 12% polyacrylamide electrophoresis gel. Proteins were electrotransferred to polyvinyldifluoride (PVDF) membranes (Immobilon-P, Millipore, Burlington, MA, USA) in Tris-glycine-methanol-buffered solution. Membranes were blocked with 5% dried skim milk in 0.1% Tween 20 and Tris-buffered saline pH 8 (TBST) for 1 h at RT, and then extensively washed in TBST. Immunodetection was performed by incubating the membranes overnight at 4 °C with the following antibodies: rabbit polyclonal (pAb) anti-SMN (1:200; Santa Cruz Biotechnology, Dallas, TX, USA; cat. #sc-15320) and mouse monoclonal (mAb) anti-SMN (1:1000; BD Transduction Laboratories^TM^, San Jose, CA, USA; cat #610646). Rabbit polyclonal anti-actin (1:5000; Sigma-Aldrich; cat #A5060), mouse monoclonal anti-glyceraldehyde 3-phosphatase dehydrogenase (GAPDH; 1:10,000; Abcam, Cambridge, UK; cat. #ab8245), and rabbit monoclonal anti-GAPDH (1:10,000; Abcam, cat. #ab181602) antibodies were used for loading controls. The membranes were washed in TBST, incubated with the appropriate peroxidase-conjugated secondary antibodies (1:20,000; Cell Signaling Technology, Danvers, MA, USA) for 60 min at RT, washed in TBST, and visualized using the Amersham ECL Select Western Blotting Detection Reagent (Cytiva, Marlborough, MA, USA), as described by the manufacturer. The quantification of band densities was performed using a Chemi-Doc MP Imaging System (BIO-RAD Laboratories Inc.).

### 2.6. Immunohistochemistry

Muscles were fixed in 4% PFA for 90 min, washed (0.1 M glycine in PBS) for 30 min, permeabilized (1% [*v*/*v*] Triton X-100 in PBS) for 90 min, and incubated in 5% (*w*/*v*) bovine serum albumin with 1% Triton X-100 in PBS for 3 h. Samples were incubated overnight at 4 °C, with the following primary antibodies: rabbit polyclonal anti-SMN H195 (sc-15320, SCBT), mouse monoclonal anti-NF-M (sc-51683, SCBT), and goat polyclonal anti-MAP1B (sc-8970, SCBT). The next day, muscles were incubated in PBS containing 0.05% Triton X-100 for 1 h, exposed to the appropriate secondary antibodies for 1 h (Alexa Fluor 647-conjugated donkey anti-goat or donkey anti-rabbit (Invitrogen, Madrid, Spain), Alexa Fluor 647-conjugated donkey anti-chicken (Jackson 703-605-155), Alexa Fluor 594-conjugated donkey anti-rabbit (Invitrogen, Madrid, Spain), CF488-conjugated donkey anti-mouse (Biotium, Madrid, Spain), plus 10 ng/mL bugarotoxin-rhodamine- (BTX-rho), and bathed again with 0.05% Triton X-100 for 90 min. Finally, muscles were mounted with Slowfade medium (Invitrogen, Madrid, Spain)).

### 2.7. Image Acquisition and Analysis

Images were acquired using an upright Olympus FV1000 multispectral confocal microscope, equipped with three excitation laser lines: (i) multiline argon laser (M-Ar) with excitation at 458, 488, and 515 nm, (ii) helium-neon green laser (HeNeG) with excitation at 561 nm, and (iii) helium-neon red (HeNeR) laser excited at 633 nm. As previously described [30], the excitation was carried out sequentially for the different lasers, to avoid cross-excitation of the samples labeled with different fluorophores. Images were acquired as serial optical sections on the Z-axis (Z-stacks) using a PlanApo N-type 60× oil immersion objective (Olympus) with a numerical aperture (NA) of 1.42. Images from wild-type and transgenic control and littermate SMA mice were taken with similar conditions (laser intensity and photomultiplier voltage) and, usually, during the same day. Only superficial nerve terminals were imaged. Fluorescence distribution and area were analyzed using ImageJ routines. Presynaptic and endplate areas were delineated with outline masks based on brightness thresholding from maximal projected confocal images. In all immunohistochemistry (IHC) experiments, endplate acetylcholine receptors were stained with BTX-rho. The ratio between the fluorescent presynaptic area of interest and its BTX-rho labeled endplate area was calculated for each NMJ. The background signal was subtracted using the same threshold used for the analysis of the presynaptic area.

### 2.8. STED Super-Resolution Microscopy

Stimulated emission depletion (STED) super-resolution images were acquired using a STEDYCON nanoscope system (Abberior Instruments) coupled to a FV1000 microscope. A 775-nm laser was used for depletion. In some cases, the images were deconvoluted with the Huygens Essenstials^®^ software and processed and analyzed using the ImageJ software (W. Rasband, NHI, Bethesda, MD, USA; http://rsb.info.nih.gov/ij/, accessed on 20 August 2022). The background signal was subtracted and smoothed before analysis. SMN granules’ size was analyzed by measuring their full-width at half maximum (FWHM) values of their intensity profiles with ImageJ (complement: Adrian’s FWHM) and estimating the area, assuming they were perfect circles. For analysis of the SMN signal, mean intensity, maximum intensity, and integrated density were measured in regions of interest potentially equivalent to individual granules. The number of SMN granules was estimated using the routine *analysis of particles*. The density of the granules was measured in individual planes that were normalized to the BTX-rho area.

### 2.9. Statistical Analysis

Statistical analysis of data was performed using GraphPad Prism 5 (GraphPad Software). Unless otherwise stated, all values mentioned in the text and represented in graphs are the mean ± standard errors of the mean (SEM). Parametric statistics were used whenever possible. The assumption of homogeneity of variances was assayed with Levene’s test, using α = 0.05 as a cut-off. When the distribution was normal, statistical comparisons between experimental conditions were made using Student’s paired two-tailed *t*-test, or unpaired *t*-test, as indicated. When the distribution was not normal, a Mann–Whitney rank-sum test was used. Given that the number of nerve terminals analyzed per condition was typically 5 or less in some of the live imaging experiments, every presynaptic terminal was treated as statistically independent. The results were considered statistically different when the *p*-value was ≤ 0.05. Data in parentheses (n, N): n, the number of nerve terminals per group; N, number of mice per group.

## 3. Results

### 3.1. SMN Is Localized as Granules in Axons and Motor Nerve Terminals

Previous studies showed that SMN is present in the axons of cultured motor neurons [8,9,31] and diaphragmatic nerve terminals [32]. Here, to further investigate the localization and organization of endogenous Smn within axons and nerve terminals, we performed IHC and quantitative confocal microscopy in neuromuscular preparations of the TVA muscle from wild-type mice at P3. The specificity of the anti-SMN pAb was validated by WB (see below) and immunocytochemistry in isolated shSMN motor neurons (Figure S1A) and spinal cord sections from control and SMAΔ7 mice (Figure S1B).

**Figure 1:**
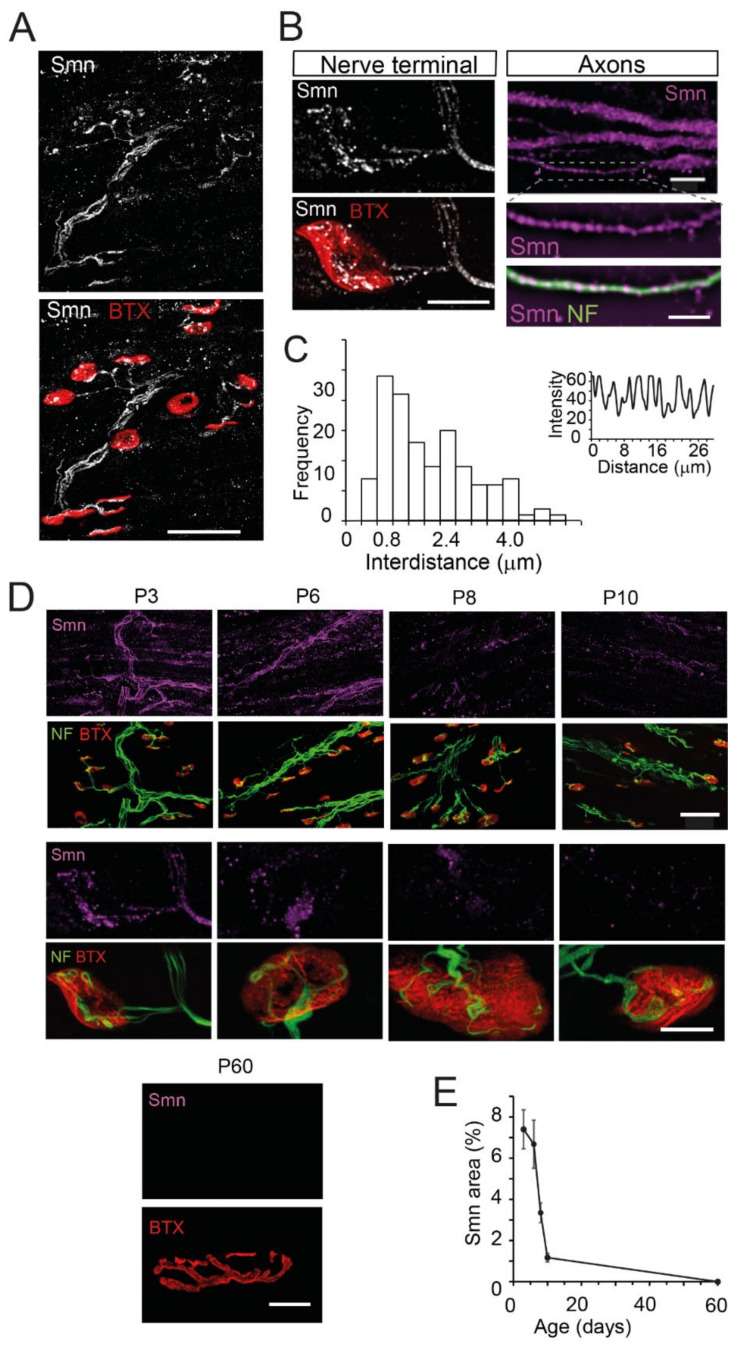
SMN has a granular appearance and progressively decreases during the second week of life in axons and motor nerve terminals of wild-type mice. (**A**). Maximum intensity projection in a representative neuromuscular preparation of the TVA muscle at P3. Immunofluorescence of endogenous SMN (Smn, white) is detected in motor axons and nerve terminals. Note the apposition of SMN with the postsynaptic marker BTX-rhodamine (red). Scale bar: 50 μm. (**B**). SMN (white) shows a granular pattern at the NMJ (left panels, scale bar: 10 μm), and axons (magenta, right panels, upper-scale bar: 5 µm, lower-scale bar: 3 µm). Note the single-file arrangement of the SMN granules in the thinner axons (inset) marked by anti-NF (green). (**C**). Frequency histogram of the spacing between axonal granules, as calculated by their intensity profile (inset). (**D**). The SMN signal (magenta) is intense at P3 and P6, weak at P8 and P10, and almost null at adulthood (P60) in the TVA muscle. Scale bars upper panels: 50 µm (P3–P10), 10 µm (P60); lower panels: 10 µm. (**E**). The graph displays the percent of the Smn area with respect to the postsynaptic area at different ages.

In the neuromuscular preparations, an intense Smn signal distributed in axons and axon terminals was observed (Figure 1A). Moreover, a weak signal was found outside the NMJ, in accordance with the presence of the Smn protein in muscle fibers [33,34]. When images were acquired with a higher magnification, the Smn signal appeared in the form of granular structures in axons and motor nerve terminals (Figure 1B). In the thinnest axons, the granules were found arranged in a single row, with a mean distance (center-center) of 1.95 ± 0.12 μm (*n* = 114 granules, 9 axons, N = 3 mice) (Figure 1C). The mean Smn density of granules in the thinnest axons was 0.55 ± 0.03 per μm (*n* = 123 granules, 9 axons, N = 3 mice).

### 3.2. Smn Progressively Decreases during the Early Postnatal Period in the Axon and the Presynaptic Motor Terminal of Wild-Type Mice

SMN levels physiologically decrease in the cell bodies of motor neurons and in various tissues during the early postnatal period in mouse models and humans, indicating a significant role of SMN during development and maturation [35,36,37,38,39,40,41]. Since defects in the NMJ maturation have been reported in SMA mouse models [30,42,43,44], we investigated whether the physiological changes in the expression of SMN observed in other tissues also occur in motor axon and nerve terminals. For this purpose, we quantified the endogenous Smn signal in the TVA muscle of wild-type mice at different ages.

During the first week of the postnatal period, the Smn signal was intense in the axons and nerve terminals (Figure 1D). At the presynaptic terminal, it represented 7.37 ± 0.61% of the postsynaptic area at P3 (38 terminals, 3 mice) and 7.18 ± 3.52% at P6 (28 terminals, 2 mice). However, during the second postnatal week, there was a significant reduction in the signal, representing a 3.34 ± 0.28 of the postsynaptic area at P8 (24 terminals, 2 mice) and 1.17 ± 0.1% at P10 (22 terminals, 2 mice) (*p* < 0.005 and *p* < 0.0005, respectively, compared to P3; one-way ANOVA, Bonferroni’s post hoc test). The Smn signal was not detectable during adulthood (P60) using the same excitation and acquisition parameters as at postnatal age. However, the signal could be detected when higher laser intensities were applied. Figure 1E shows the measured time course of Smn reduction at the presynaptic terminals in wild-type mice.

### 3.3. β-Actin mRNA Transcripts Localize in the Presynaptic Compartment

The spatiotemporal decrease of endogenous Smn in wild-type mice during the early postnatal period indicates a role of Smn in the early postnatal maturation of motor nerve terminals. In accordance with this, it has recently been shown that Smn-specific mRNAs are associated with neurogenesis and translation [45]. In neuronal cells with distant synaptic targets, local protein synthesis has been proposed to be important in developing and mature axons, to regulate the proteome of cellular subcompartments [46,47,48,49,50,51]. Since SMN granules in vitro contain mRNAs and RNPs, we postulated that SMN participates in local protein translation *in vivo*. To test our hypothesis, we investigated the presence of mRNA transcripts in axons and nerve terminals in whole-mount TVA muscles from wild-type (P3–P6) mice by fluorescence in situ hybridization (FISH) [52].

As *β-actin* mRNA is abundant in neurons [53,54], FISH experiments were carried out using an mRNA-binding probe for *β-actin* conjugated to an immunolabeled DIG polypeptide. In these experiments, protein digestion is needed, to dissociate the RNA–protein complexes and make the RNA of interest accessible to the probe. However, if protein digestion is excessive, identifying structures by marker proteins, such as cytoskeletal proteins or acetylcholine nicotinic receptors, can be compromised. Thus, we initially tested different experimental conditions, to obtain the optimal enzymatic digestion of the tissue, without altering the detection of the marker proteins. We tested 10 and 2 mg/mL of proteinase K with different incubation periods (5–45 min). Although the characteristic dotted signal of mRNA labeling [18,55] was obtained with 10 mg/mL of proteinase K, the marker protein (Tau) could not be adequately identified, indicating over-digestion. With 2 mg/mL of proteinase K, an adequate signal from the RNA probe was obtained at all times tested (Figure S2A, upper panels), though the marker protein (Tau) signal was seen only with 5 min of incubation (Figure S2A, lower panels). Thus, we incubated the muscle preparations with 2 mg/mL proteinase K for 5 min during the rest of the study. Additionally, we performed two control experiments to check whether there was bleed-through or crosstalk between the DIG-488 nm and α-bungarotoxin (BTX)-Cy3 channels. The results showed that the emission of the green channel did not pass through the red channel (Figure S2B, upper panels), and the signal from Cy3 did not cross to the green channel despite its high intensity (Figure S2B, lower panels). Finally, we checked the specificity of the anti-DIG antibody, by performing a negative control experiment in the absence of the *β-actin* mRNA binding probe. No specific signal was obtained in those cases (Figure S2C, left panel), while the NMJs were identified by the immunostaining of Tau (Figure S2C, right panel).

**Figure 2:**
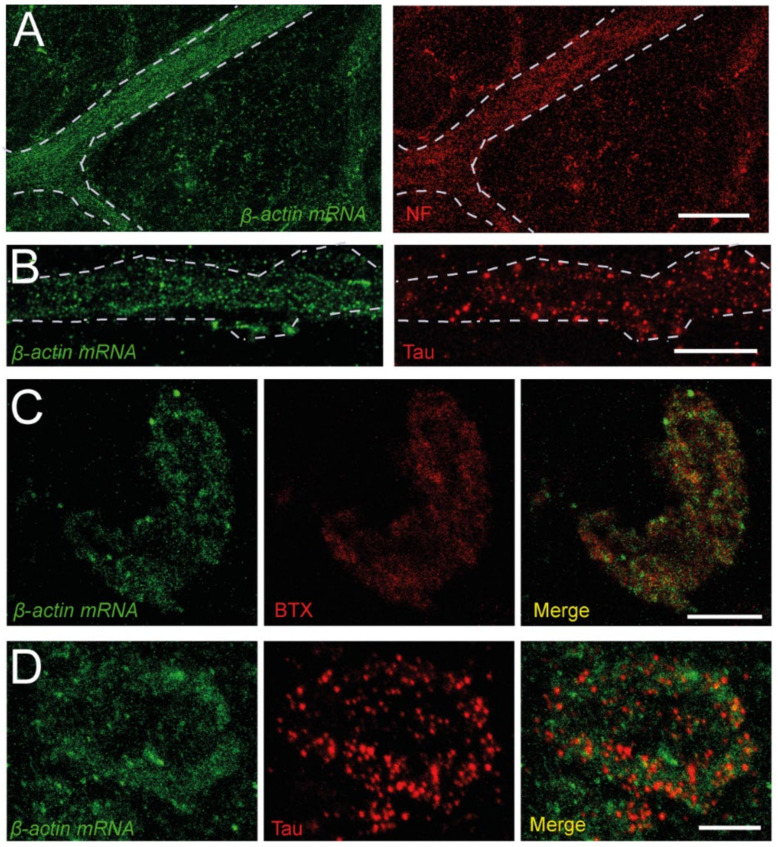
*β-actin* mRNA is present in peripheral axons and NMJs. (**A**). Example of *β-actin* mRNA (green) in a bundle of axons visualized by FISH (left panel). The same axons are marked with an anti-NF antibody (red, right panel). (**B**). Detail of the dotted signal of the probe in a band of axons (left panel), identified by its immunoreactivity to Tau (red, right panel). (**C**). Images of an NMJ displaying *β-actin* mRNA (green, left panel) and BTX (red, middle panel), and a merged image (right panel) (**D**). Images of an NMJ displaying *β-actin* mRNA (green, left panel) and Tau (red, middle panel) signals, and a merged image (right panel). Calibration bars: (**A**) 40 µm, (**B**–**D**) 10 µm.

Once the enzyme digestion conditions and antibody specificity were established, we investigated whether the *β-actin* mRNA localizes at the presynaptic compartment, besides its presence in axons. Figure 2 shows that the *β-actin* mRNA signal is located close to NFs and Tau in axons (panels A and B, respectively). Remarkably, a similar signal for the transcript was also found in correspondence with the labeling of acetylcholine receptors and Tau at the NMJs (Figure 2C and Figure 2D, respectively), showing, for the first time, the presence of *β-actin* mRNA at the NMJ presynaptic compartment.

### 3.4. Ribosomes and Polysomes Are Present in Nerve Terminals

**Figure 3:**
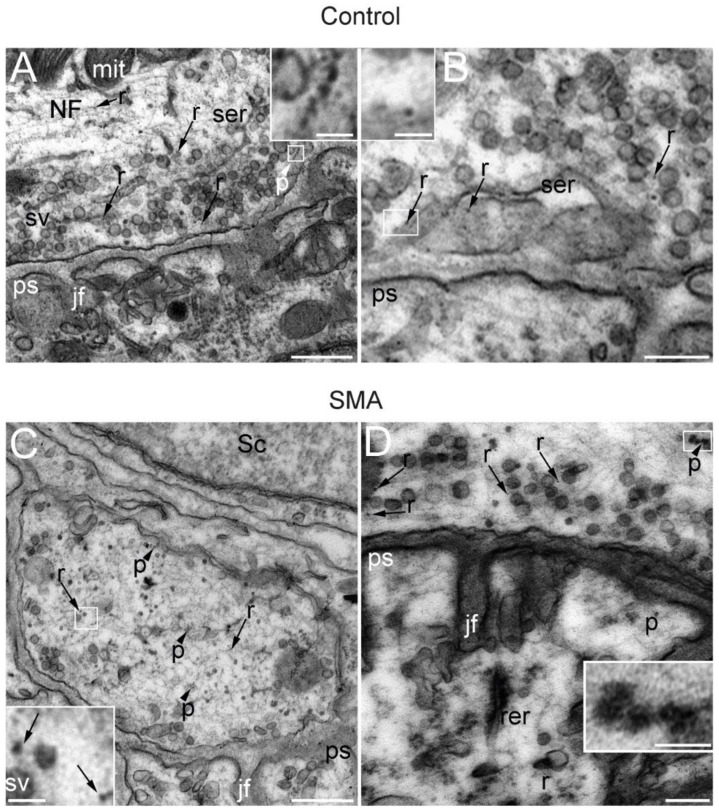
SMA and control presynaptic motor terminals contain ribosomes and polysomes. Electron microscopy representative images of NMJs from the TA muscle of control (**A**,**B**) and SMA (**C**,**D**) mice from the SMNΔ7 line at P14. In the cytoplasm of the presynaptic terminal, synaptic vesicles (sv), neurofilaments (NFs), mitochondria (mit), and smooth endoplasmic reticulum (ser) are indicated. Scattered throughout the cytoplasm of the presynaptic terminal are numerous independent ribosomes (r) (arrows and boxes in **B**,**C**) and, less abundantly, polysomes (p) (arrowheads and boxes in **A**,**D**). Other identified elements incude jf: junction folds; ps: postsynaptic element; rer: rough endoplasmic reticulum; Sc: Schwann cell. Calibration bars: 500 nm (**A**,**C**), 200 nm (**B**,**D**), and 50 nm (inserts from **A**,**D**).

Aside from the role of SMN in mRNA translocation to the peripheral subcellular compartments, it has recently been shown that SMN associates to polysomes and participates in translation in cultured motor neurons [21], murine brain, and spinal cord samples [56]. Furthermore, in vivo experiments have shown that SMN directly modulates a specific subset of ribosomes implicated in the translation of mRNAs related to ribosome biogenesis, bioenergetics, and neuronal function [45]. Ribosomes have been identified at motor nerve terminals by immunoelectron and conventional electron microscopy [57,58]. To check for the presence of ribosomes at the presynaptic motor terminal of control and SMA SMNΔ7 mice, we performed a transmission EM study and investigated the distribution of ribosomes and polysomes, according to the criteria of these previous studies. Figure 3 shows representative examples of NMJs in TA muscles at P14. In both the control (Figure 3A,B) and SMA mice (Figure 3C,D), abundant dispersed ribosomes, as well as polysomes, were seen at the presynaptic terminal (arrowheads and boxes). Since we had already found SMN and mRNA transcripts at the preterminal compartment, the presence of ribosomes demonstrates that motor nerve terminals in control and SMA mice contain the molecular machinery necessary for protein synthesis, and this gives additional support to the occurrence of in vivo local translation in this compartment. Furthermore, the presence of polysomes strongly suggests that active local translation is taking place during the NMJ postnatal maturation period in both genotypes. Nevertheless, in SMA mice, some terminals showed abundant degenerated mitochondria, lysosomes, and autophagosomes (Figure S3), indicating that the neurodegeneration process was also highly activated.

### 3.5. Axonal and Presynaptic SMN Granules Abundancy Remains High in SMA Models

Control and SMA mice of the widely used SMNΔ7 line contain two copies of the human SMN2 and several copies of the *SMNΔ7* cDNA, with transgenic control mice also expressing the murine *Smn* gene [27]. We investigated whether the transgenic SMN protein exhibited a similar spatial distribution to the endogenous Smn protein. To address this question, we studied the SMN expression in SMNΔ7 mice at P9-10, an age at which the endogenous Smn signal in wild-type mice had already decreased by 87% (Figure 1E).

**Figure 4:**
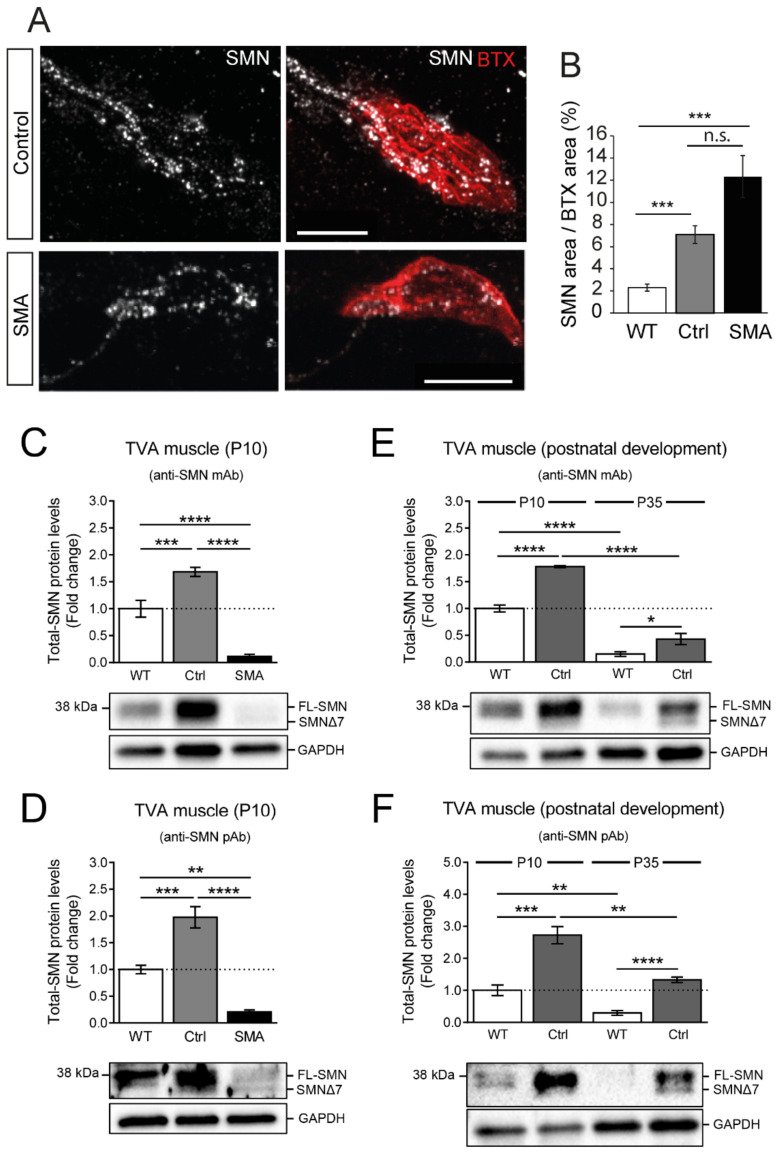
SMN signal was high in motor synaptic terminals of control and SMA mice at P9-10. (**A**). Maximum intensity projections of TVA muscle motor terminals in both genotypes at P9. The motor endplates were identified using BTX-rhodamine labeling (red). Scale bars: 10 µm. (**B**). Comparison of the SMN area normalized to the postsynaptic area (BTX) in wild-type and SMNΔ7 mice (control and SMA). The analysis was performed at P9-10. ***: *p* < 0.01; n.s.: not significant; (**C**,**D**). Quantification and representative images of Western blot assays performed on TVA muscle extracts obtained from P10 wild-type (WT) and transgenic SMNΔ7 mice (control (Ctrl) and SMA). Either a mouse monoclonal antibody (mAb) anti-SMN or a rabbit polyclonal antibody (pAb) anti-SMN was used in the analysis. Note that with both the anti-SMN mAb (**C**) and the pAb (**D**), total-SMN levels were significantly increased in Ctrl, compared to WT (***: *p* < 0.001). In contrast, relative expression of total SMN in SMA mice was dramatically reduced compared with either WT or Ctrl animals (****: *p* < 0.0001 vs. WT or Ctrl (with the anti-SMN mAb); **: *p* < 0.01 vs. WT and ****: *p* < 0.0001 vs. Ctrl (with the anti-SMN pAb)). In all the cases, total-SMN levels (FL-SMN and SMNΔ7, when present) were quantified in 4 WT and in 8–11 Ctrl and SMA mice and normalized; first, to the loading control (GAPDH), and then, to the average of the WTs. Data are presented as mean ± SEM and were analyzed by using a one-way ANOVA (Bonferroni’s post hoc test). (**E**,**F**). Quantification and representative images of Western blot assays performed on TVA muscle extracts obtained from P10 and young adult (P35) WT and Ctrl transgenic SMNΔ7 mice, using either the anti-SMN mAb or pAb. Note that, with both the anti-SMN mAb (**E**) and the pAb (**F**), total-SMN protein levels at P35 were significantly lower than at P10, in WT and Ctrl mice (****: *p* < 0.0001 vs. P10 WT or Ctrl (with the anti-SMN mAb); **: *p* < 0.01 vs. P10 WT or Ctrl (with the anti-SMN pAb)). Moreover, at P35, SMN protein levels in TVA muscles remained significantly higher in Ctrl than in WT (*: *p* < 0.05 vs. WT (with the anti-SMN mAb); ****: *p* < 0.0001 vs. WT (with the anti-SMN pAb)). In all cases, total-SMN levels (FL-SMN and SMNΔ7, when present) were quantified in 4 mice per experimental condition and normalized; first, to the loading control (GAPDH), and then, to the average of the P10 WTs. Data are presented as mean ± SEM. Each two conditions were compared using a two-tailed Student’s *t*-test.

Unexpectedly, abundant SMN granules were identified in control and SMA SMNΔ7 axons and nerve terminals (Figure 4A). For quantification, the area of the SMN signal was normalized to the postsynaptic area in both genotypes and compared with that in wild-type mice (Figure 4B). The SMN signal values were 7.05 ± 0.8% in transgenic control (*n* = 38 terminals, 3 mice) and 12.41 ± 1.91% (*n* = 32 terminals, 3 mice) in SMA mice. These values were significantly different from those obtained at a similar age (P8-10) in wild-type mice, representing a 3.1-fold and 5.5-fold increase, respectively (*p* < 0.0001, Kruskal-Wallis test; Dunn’s post hoc test versus wild-type, *p* < 0.0001 in both genotypes).

Axons of control and SMA mice also showed an intense SMN signal and a granular pattern (Figure S4A, left panels). The SMN content varied, depending on the thickness of the axon, but was much higher in transgenic mice than in wild-type mice at P8-10 (compare Figure 1D and Figure S4A, left panels). We also checked for the presence of SMN in the axons of the levator auris longus (LAL) muscle (Figure S4A, right panels), which is less morphologically and functionally affected than the TVA in SMA models [59,60,61,62,63], and found a similar granular content to that in the TVA axons.

### 3.6. SMN Protein Levels Are Elevated in the TVA Muscle of Control Transgenic Mice

After observing that motor nerve terminals of both control and SMA transgenic SMN∆7 mice displayed a higher abundancy of SMN-containing granules than wild-type animals, we further investigated the possible differences in SMN expression between these conditions.

We first examined SMN protein levels in the entire TVA muscle from P10 wild-type and transgenic SMNΔ7 mice using Western blot. To increase the feasibility of detecting the total SMN protein, we performed, in parallel, independent experiments using two widely used anti-SMN antibodies: a mouse mAb, and a rabbit pAb. We found that at P10, the relative levels of total SMN protein in transgenic controls were significantly higher compared with those in wild-type muscles. This increase was observed regardless of the antibody used for SMN detection (fold increase: anti-SMN mAb, 1.68 ± 0.09 (*p* = 0.0003), and anti-SMN pAb, 1.98 ± 0.2 (*p* = 0.0006); one-way ANOVA, Bonferroni’s post hoc test) (Figure 4C and Figure 4D, respectively). These data are in agreement with the increased immunolabeling observed in motor nerve terminals of transgenic control compared to wild-type mice (see Figure 4B).

In contrast, and as expected, the relative expression of total SMN in SMA mice was dramatically reduced compared with either wild-type or transgenic control animals (Figure 4C,D). These results are consistent with previous data reporting a decline in SMN protein expression in the muscles of SMA mice, but differ from the increased immunolabeling of SMN protein we observed in presynaptic motor terminals of SMA mice compared to wild-type (see Figure 4B). These divergent results could be ascribed to the scarce representation of pre-synaptic motor terminals in the total TVA muscle extracts used in our Western blot analysis. Thus, the increased SMN expression we observed in SMA motor nerve terminals was probably masked by the reduced SMN levels occurring in SMA muscle cells.

Next, we analyzed whether this differential SMN expression found between transgenic control and wild-type mice at P10, persisted during young adulthood. To assess this, we compared SMN levels in control and wild-type mice at P35. To minimize the inter-experimental variability, new muscle samples at P10 were also included in the study. At P35, SMN protein levels in the TVA muscle were significantly lower than at P10, in either wild-type or control mice (Figure 4E,F). This agrees with the previously described reduction in SMN protein levels after maturation of the neuromuscular system [40,41]. However, even at this age (P35), TVA muscles from transgenic control mice continued to exhibit higher levels of total SMN protein than those from wild-type animals (fold increase: anti-SMN mAb, 2.86 ± 0.69 (*p* = 0.0484), and anti-SMN pAb, 4.52 ± 0.29 (*p* < 0.0001); Student’s *t*-test, two-tailed) (Figure 4E and Figure 4F, respectively).

Finally, we wanted to examine whether similar differences in SMN expression between wild-type, control, and SMA transgenic mice also occurred in the OA muscle and in the spinal cord. Since the anti-SMN mAb and pAb followed a similar pattern of SMN protein detection in TVA muscle, only the mAb was included in the analysis. As expected, we found that in both the OA muscle and spinal cord from P10 SMA animals, SMN levels were significantly reduced compared to the respective wild-type or control samples. However, in the samples from transgenic control animals, SMN levels were, again, substantially higher than in those from wild-type mice (fold increase in OA muscle: 1.95 ± 0.15 (*p* < 0.0001); fold increase in the spinal cord: 1.75 ± 0.26 (*p* = 0.0442); one-way ANOVA, Bonferroni’s post hoc test) (Figure S5A and Figure S5B, respectively). At P35, as expected, the SMN protein levels in both tissues were significantly lower than at P10, in both wild-type and control. However, in OA muscles, the SMN levels in control animals remained significantly higher compared to the wild-type mice (Figure S5C) (fold increase: 2.31 ± 0.22 (*p* = 0.0034, Student’s *t*-test, two-tailed)), as we previously found in the TVA muscle (Figure 4E,F). Nevertheless, in the spinal cord, the SMN levels were not statistically different between control and wild-type (fold increase: 1.1 ± 0.15 (*p* = 0.5425, Student’s *t*-test, two-tailed)) (Figure S5D).

Overall, these results show that the SMN protein levels were significantly higher in the transgenic control than in wild-type mice, probably due to the SMN overexpression resulting from the introduction of two human *SMN* transgenes.

### 3.7. SMN Granules Visualized at Super-Resolution

**Figure 5:**
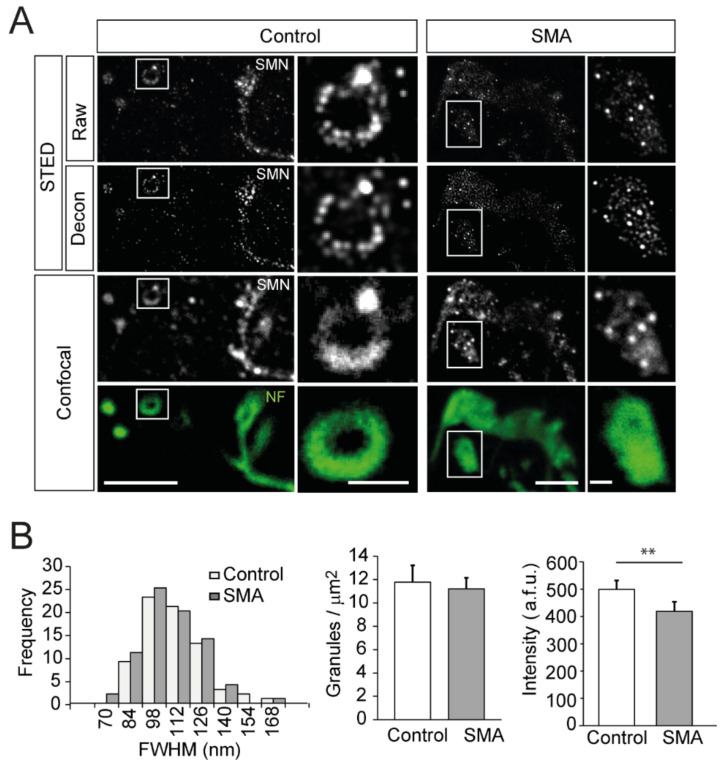
SMN granule properties visualized at super-resolution. (**A**). Comparison of immunofluorescence signals from SMN (white) with super-resolution and confocal microscopy in motor nerve terminals of the TVA muscle at P9. The super-resolution signals after deconvolution are also shown. The intraterminal axonal branches were labeled with antibodies against NFs (green). Note the ring-like (control) and the plaque-like (SMA) structures of the final part of the NFs branches and the ordered organization of granules in control. Scale bars: 5 μm and 1 μm (insets). (**B**). The apparent size distribution of SMA granules is similar in the control and SMA mice (left graph). The density of granules in the axonal branches is similar in both genotypes (middle graph). The mean granule intensity is lower in SMA mice (right graph; **: *p* < 0.005). a.f.u., arbitrary fluorescent units.

We used super-resolution STED microscopy to increase the size measurement accuracy of the granules and their distribution in the presynaptic motor terminal. The system consisted of a compact STEDYCON unit (Abberior) coupled to our FV1000 confocal microscope. The analysis of the STED images in the P9 TVA muscle from control and SMA samples of the SMNΔ7 model confirmed the granular pattern of the immunostaining and allowed a better characterization of the granules. Representative examples are shown in Figure 5A, where STED and conventional confocal images are compared in both mouse genotypes. Identifying individual granules was much better with STED, and the images were further improved when a deconvolution routine (Huygens) was applied. Typically, a rosette-like organization of the granules within the final part of the NFs was observed in control mice. In contrast, plaque-like structures were most frequently observed in SMA animals (Figure 5A, insets).

The analysis of the diameter of the granules with super-resolution was carried out in the same way as in conventional confocal microscopy. The average size of the granules was similar between control and SMA mice (Figure 5B, left graph) (*p* = 0.3; Student’s *t*-test, two-tailed), with a mean value of 103.4 ± 2.32 nm (73 granules) and 100.31 ± 1.93 nm (77 granules), respectively, coinciding with the optical resolution that we obtained in the experiments. Likewise, the apparent mean density of granules in regions where they displayed a rosette- or a plaque-like organization was similar in both genotypes (Figure 5A, insets), with a mean value of 12 granules/µm^2^ (Figure 5B, middle graph). We found significant differences in the mean intensity of granules between genotypes (Figure 5B, right graph; *p* = 0.0021, two-tailed Mann-Whitney U test), with mean values of 499.3 ± 32.13 arbitrary fluorescent units (a.f.u.) in control (73 granules) and 418.9 ± 34.41 a.f.u. in the SMA (77 granules) mice. Remarkably, the mean intensity of granules did not correlate with their diameter (Figure S6A; R^2^ = 0.08 and 0.019 in control and SMA, respectively).

### 3.8. SMN Is Spatially Associated with MAP1B in SMA and Controls

The integrity and dynamics of the cytoskeleton are essential for the proper function of axons and synaptic terminals. SMN interacts with the proteins that regulate the cytoskeleton dynamics, such as profilin, an actin-binding protein [64,65,66]. Microtubules, which participate in the maintenance of the cell structure and transport of organelles, are the largest and most dynamic filaments of the cytoskeleton and are impaired in SMA [62,67]. MAP1B, a microtubule stabilizer protein that participates in the neuronal development of axons, growth cones, and presynaptic terminals [68,69], is also altered in SMA models [70] (https://idus.us.es/handle/11441/40896, accessed on 20 August 2022).

**Figure 6:**
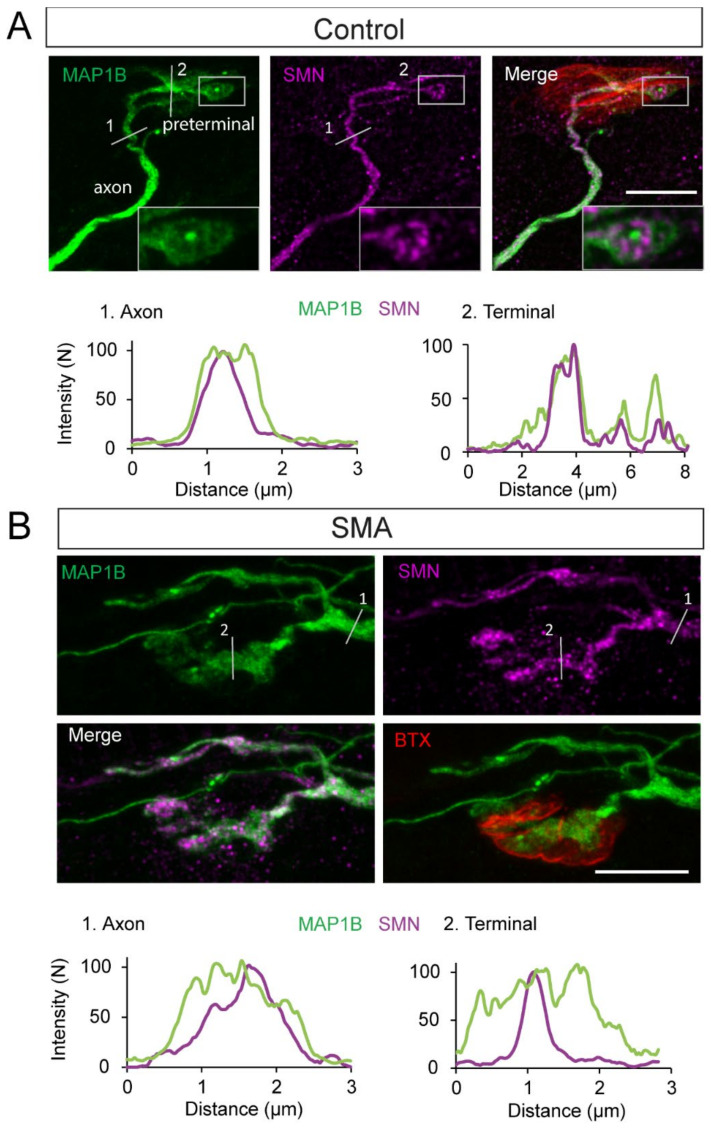
SMN granules are spatially associated with MAP1B at presynaptic motor terminals. (**A**). SMN granules (magenta) follow the MAP1B trajectory (green) in control mice, both in the axon and the presynaptic terminal. MAP1B can form rounded structures containing organized SMN granules (inset) at the most distal portions of the intraterminal branches. The linear intensity profile of MAP1B (green) and SMN (magenta) in segments 1 and 2 in the images are shown in the lower graphs. (**B**). In SMA mice, MAP1B has a diffuse appearance. Linear intensity profile of MAP1B (green) and SMN (magenta) in segments 1 and 2 indicated in lower graphs. Note that the MAP1B signal encompasses the SMN signal. Scale bars: 10 μm.

Since axonal SMN associates with the microtubule apparatus, as has been shown by EM immunolabeling [71], we hypothesized that this relationship should also be found at the level of the presynaptic terminal. Thus, we explored the spatial correlation between SMN granules and MAP1B by double immunolabeling in the Taiwanese SMA model, which displays an intraterminal distribution of SMN granules similar to that found in the SMNΔ7 model (Figure 6).

SMN granules and MAP1B in control mice (*Smn^+/−^; SMN2^+/0^*) displayed almost overlapping spatial distributions, both in the axons and presynaptic terminals. However, in the most distal portions of the intraterminal branches, MAP1B formed rounded structures containing rosette-like assemblies of SMN granules (Figure 6A, inset). In SMA mice (*Smn^−/−^; SMN2^+/0^*), the MAP1B signal also coincided with the SMN signal, but both exhibited more diffused distributions than in controls (Figure 6B). These results suggest that both proteins are in close association, and motor proteins potentially transport SMA granules. This hypothesis is reinforced by a previous report that found bi-directional active trafficking of axonal SMN-containing granules in cultured motor neurons [72].

### 3.9. SMN and NFs Follow the Same Path at Motor Nerve Terminals

**Figure 7:**
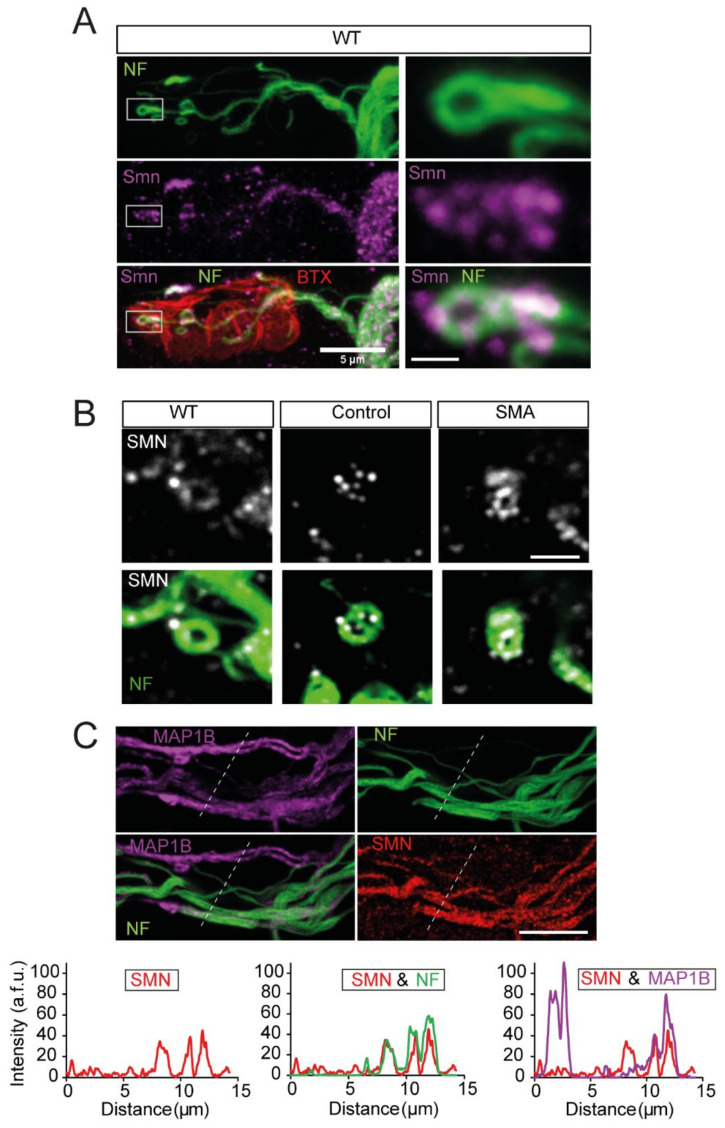
SMN granules co-localize with NFs in presynaptic motor terminals and axons. (**A**). Representative example showing that SMN granules (magenta) follow the NF (green) path, from the motor nerve to the distal part of the intraterminal branches, in a TVA muscle from a wild-type mouse. The endplate was labeled with BTX (red). On the right panels, the delimited regions are shown magnified. Scale bars: 5 µm and 750 nm, respectively. (**B**). Details of SMN granules (grey), following NFs intraterminal structures (green) in wild-type and SMNΔ7 (control and SMA) mice. Scale bar: 2 µm. (**C**). SMN granules show a greater degree of coincidence with the NFs than with MAP1B. Example of a band of axons from a Taiwanese control mouse labeled for MAP1B (magenta), NFs (green), and SMN (red). Note the heterogeneity of the axons, in terms of their immunoreactivity for each of these proteins. Scale bar: 10 µm. The graphs display the intensity profiles through the lines in the images. Note the better coincidence of SMN (red trace) with axons showing a high NFs intensity (green traces) than with axons displaying a high MAP1B content (magenta traces). a.f.u., arbitrary fluorescent units.

In wild-type mice, at early postnatal ages, NF showed a branched arrangement ending in loop-like structures in motor nerve terminals (Figure 7A, upper panels). The spatial distribution of the endogenous Smn granules (Figure 7A, middle panels) was consistent with that of the NFs (Figure 7A, lower panels), indicating that the Smn distribution closely follows NFs. At SMNΔ7 mouse presynaptic terminals, the relationship between NFs and SMN granules was conserved, as revealed by the concentration of SMN granules at NFs loops (Figure 7B). These data confirm a previous report which showed that SMN immunoreactivity is associated with NFs [71].

Thus, given that SMN granules showed an apparent association with NFs and MAP1B, we next studied the spatial relationship between these three proteins in axons. The triple immunolabeling for MAP1B, NF, and SMN revealed that the SMN granule content correlated well with both cytoskeletal proteins (Figure 7C), except in some cases where MAP1B-marked axons displayed no NFs or SMN granules (Figure 7C, right graph), suggesting a closer association of SMN granules with NFs than with MAP1B.

### 3.10. NFs and SMN Aggregate Together

The pathological abnormality of NFs in SMA has been well documented [43,44,59,61,62,63,73]. Hence, to better understand the involvement of NFs in SMA pathophysiology, we studied the relationship between NFs and SMN granules at the nerve terminals and axons displaying a certain amount of NFs accumulation.

**Figure 8:**
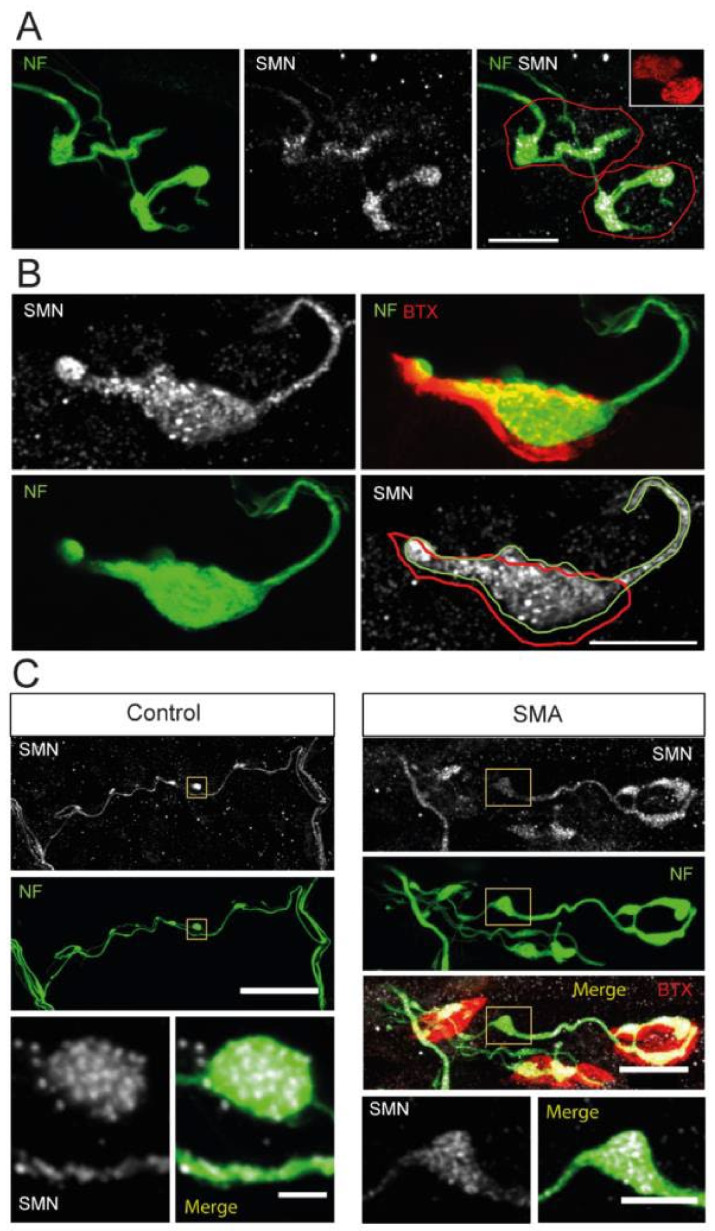
SMN aggregates in NF accumulations at P9. (**A**). Representative example of motor synaptic terminals (red outlines) displaying few NFs branches (green) and aggregates of SMN granules (white) in an SMA mouse. Scale bar: 10 µm. (**B**). SMA presynaptic terminal showing a massive accumulation of NFs (outlined in green), occupying much of the terminal area (red outline) and containing abundant SMN granules (white). Scale bar: 10 µm. (**C**). Axonal accumulations of NFs in the SMNΔ7 model coinciding with SMN granule aggregates, both in transgenic control and SMA mice. The insets show details of the overlap of SMN granules and NFs. Accumulations of SMN granules and NFs were only occasionally observed in control mice. The scale bars are 40 and 2 µm (left panels) and 15 and 5 µm (right panels).

Typically, SMA SMNΔ7 mice displayed thinner motor axons than control animals, together with significant NFs accumulations along a single intrasynaptic axonal branch (Figure 8A, left panel). We found that the SMN signal closely followed the NFs trajectory inside the presynaptic motor terminal (Figure 8A, central and right panels). Moreover, in approximately 20% of NMJs of SMA mice, a single and massive accumulation of NFs, accompanied by a high density of SMN granules, filled most of the presynaptic terminal (Figure 8B). These compact, large NF-SMN structures found in the SMA model probably represented one of the final degeneration stages of the preterminals.

As observed in nerve terminals, NFs accumulations were frequently found in axons of SMA mice, which also contained numerous SMN granules (Figure 8C, right panels). The NF-SMN aggregates were noticed both far from (Figure 8C), and close to, the presynaptic motor terminal (Figure S6B, arrows). In control mice, axonal NFs cumuli were occasionally found, but, remarkably, they also contained numerous SMN granules (Figure 8C, left panels). Together, these results indicate that SMN granules follow the NFs distribution in both physiological and pathological conditions.

## 4. Discussion

The role of SMN in the assembly of the spliceosome and the biogenesis of ribonucleoproteins has been well defined [6]. In addition, SMN is involved in axonal mRNA trafficking and local translation in cultured neurons [74,75]. However, the physiological function and pathological role of SMN in motor nerve terminals are still not well understood. Here, we investigated the expression and distribution pattern of SMN proteins, both endogenous and transgenic, in axons and presynaptic motor nerve terminals during postnatal maturation in wild-type mice and SMA mouse models. The most relevant findings in the current study are as follows: first, SMN protein appears in the form of granules, and the number of axonal and presynaptic granules decreases progressively during the second postnatal week, until they become imperceptible in adulthood; second, ribosomes and *β-actin* mRNA, two key elements of the protein synthesis machinery, are present at presynaptic terminals during the postnatal maturation stage; third, the expression of transgenic *SMN* genes leads to the persistence of SMN granules in axons and motor nerve terminals in SMA models (SMNΔ7 and Taiwanese); and fourth, NFs accumulations contained a large number of SMN granules in the presynapse and axons of SMA mice, which might contribute to the pathology of the presynaptic motor terminal.

### 4.1. Physiological Age-Dependent SMN Expression in Nerve Terminals

SMN protein levels have been documented in different tissues and ages in mouse models and human samples and found to be developmentally downregulated [35,36,37,38,40,76]. However, no data are available regarding the presence of SMN in motor axons and presynaptic terminals, except for a report on the mouse diaphragm [32]. Here, by using STED microscopy, we found that SMN is contained in the granules of 100 nm or less in diameter distributed along the axons and presynaptic terminals of TVA muscles (Figure 5A). In wild-type mice, SMN granules show a progressive temporal decrease during the second postnatal week (Figure 1D), indicating that the potential peripheral function of SMN decreases/disappears after the maturation of the presynaptic terminal. Strikingly, SMN granules persist at P9-P10 in transgenic control and SMA mice. Several explanations could account for this phenomenon. First, since the maturation of the presynaptic motor terminal is delayed in SMA mice, it could be argued that the SMN retrieval from the presynaptic motor terminal is retarded; however, in transgenic control mice, NMJs mature normally and still have a high SMN granule content. Second, in the motor neurons of SMNΔ7 mice, SMN transgenes may be differently than the mouse Smn gene. Third, a single SMN granule may contain a mix of FL-SMN and SMNΔ7 proteins, which alters the granule’s life span. SMN can form oligomers with itself [76,77], in the same way that SMNΔ7 does, although less efficiently [76]. Additionally, both isoforms can form hetero-oligomers [76,78,79,80]. Recently, three residues of the YG box (hsY277/dm208/spA141), present in both forms of the SMN protein, have been identified as major determinants for higher-order oligomerization [81]. In cultured cells, FL-SMN and SMNΔ7 heterotypic complexes increase the stability of the SMNΔ7 protein [27]. Thus, it would be of interest to determine whether the expression of the truncated protein affects, positively or negatively, the SMN granule cycle and function at motor nerve terminals.

### 4.2. The Physiological Role of SMN Granules at Motor Axons and Presynaptic Terminals

SMN-deficient motor neurons in culture exhibit significant growth defects [31], suggesting that SMN regulates axonal elongation. Although the mechanism responsible has not been well established, axonal SMN granules contain mRNAs and RNPs, which are implicated in growth, differentiation, and maturation [11,12,13,14,15,16,18,19]. Besides the possible role of SMN in mRNA transport along axons, it has been shown in vitro and in vivo that SMN interacts with a subset of ribosomes and mRNA in different tissues related to synaptic protein translation [21,45,56]. We found a progressive reduction of SMN granules at motor nerve terminals after the first postnatal week and a weak SMN signal in adult wild-type mice, suggesting a role of SMN in maturation. In addition, our FISH and electron microscopy studies revealed the presence of *β-actin* mRNA transcripts, ribosomes, and polysomes in synaptic terminals, supporting the existence of mRNA translation at the presynaptic compartment. However, detailed research on SMN-ribosome interaction and the mRNAs associated with the SMN–ribosome complex needs to be performed, to confirm the SMN function in the nerve terminals.

### 4.3. SMN Granules and Cytoskeleton

NFs accumulation in presynaptic motor terminals is a hallmark in SMA pathology [44,63,73]. Notably, our study revealed co-localization of SMN with NFs in wild-type, transgenic control, and SMA mice. We found massive NFs accumulations containing large amounts of SMN granules at the presynaptic terminals of SMA mice, probably because the SMN retrograde trafficking and homeostasis of the presynaptic compartment are compromised; this may also explain the similar amounts of SMN found in the control and SMA terminals. Further work is needed, to reveal the composition of the SMN complexes in axons and synaptic terminals and their possible role in neuronal development and maturation.

### 4.4. SMN Granules Accumulation Can Contribute to SMA Pathology

Growing evidence signals that SMA pathology is related to an SMN-dependent deficiency in forming snRNP and mRNP transport granules [74,82], affecting growth, maturation, and maintenance of different cells, particularly motor neurons. In addition, SMN deficiency decreases the ability to form stress granules, sensitizing cells to stress and promoting cell death [83]. In agreement with this, methods that successfully restore the ability of cells to form RNP granules should, in theory, alleviate the disease. In this regard, SMN gene therapy, and treatments with small molecules directed to increasing the splicing efficiency of SMN2 exon 7, have been enormously successful [84,85,86,87]. However, what are the consequences of the SMN and SMNΔ7 overexpression? It has been reported that the overexpression of SMN in scAVV9-SMN-treated control mice produces SMN aggregates in the cytoplasm of spinal motor and sensory neurons, and mice develop a sensorimotor pathology, including denervation of the NMJ [88]. In contrast to wild-type mice, SMN granules do not disappear during the second postnatal week in transgenic mice, which could be considered advantageous. However, what is the impact of increasing SMN granules in a context where NFs accumulation impairs axonal trafficking? First, if SMN granules serve to mask mRNAs until they are locally needed, SMN aggregates may prevent transcript release, contributing to the disease pathogenesis. Second, SMN aggregates may further impair the bidirectional trafficking of organelles (i.e., synaptic vesicles, mitochondria, etc.) and proteins (i.e., actin, tubulin, etc.) in nerve terminals. In various neurological disorders, including fragile X syndrome and amyotrophic lateral sclerosis, deregulated axonal mRNA trafficking and translation are linked to the disease pathology [89].

## 5. Conclusions

Our findings shed light on the expression level and distribution of SMN in the axons and synaptic terminals in physiological and pathological conditions. The present study demonstrates the existence of elements of the protein synthesis machinery at the presynaptic terminal, describes the time course of the physiological reduction of SMN granules, and reveals pathological SMN aggregation in the presynaptic motor terminal of SMA mice. We suggest that SMN is part of the local translation machinery and participates in the maturation of motor nerve terminals. In addition, our study underlines the need to consider the possible toxicity of SMN accumulation when its spatiotemporal expression surpasses the physiological levels. Thus, new tools for controlling SMN expression should be considered relevant in SMA therapy. Overall, our study creates an opportunity for future studies to better understand the physiopathological function of SMN at the motor nerve terminals.

## Data Availability

Requests for raw data or images used in the present study should be directed to the corresponding author.

## Supplementary Materials

The following supporting information can be downloaded at: https://www.mdpi.com/article/10.3390/biom12101524/s1, Figure S1: Validation of the anti-SMN H-195 antibody. Figure S2: Establishment of experimental conditions for the detection of *β-actin* mRNA in neuromuscular preparations of TVA muscles. Figure S3: SMNΔ7 mutant and control presynaptic motor terminals contain lysosomes and autophagosomes. Figure S4: SMN granular pattern in the axons of control and SMA mice of two different muscles (TVA and LAL) at P9. Figure S5: SMN protein levels in the OA muscle and the spinal cord of wild-type and transgenic SMNΔ7 mice. Figure S6: SMN size and cumuli.

## Abbreviations

AChR—Acetylcholine receptor
ANOVA—Analysis of variance
BTX—Bungarotoxin
BTX—rho-Bungarotoxin-rhodamine
Crtl—Control
DIG—Digoxigenin
EDTA—Ethylenediaminetetraacetic acid
FISH—Fluorescent in situ hybridization
FL—Full length
FWHM—Full-width at half maximum
GAP43—Growth Associated Protein 43
GAPDH—Glyceraldehyde 3-phosphatase dehydrogenase
hnRNP R—Heterogeneous nuclear ribonucleoprotein R
IHC—Immunohistochemistry
IMP1—IGF2 mRNA-binding protein 1
LAL—Levator auris longus
LNA—Locked nucleic acid
mAb—Monoclonal antibody
MAP1B—Microtubules-associated protein 1B
mRNP—Messenger ribonucleoprotein
NF—Neurofilament
NMJ—Neuromuscular Junction
OA—Obliquus abdominis
pAb—Polyclonal antibody
PCR—Polimerase Chain-Reaction
PBS—Phosphate-Buffered Saline
PFA—Paraformaldehyde
PLP—Paraformaldehyde lysine phosphate
RIPA—Radioimmunoprecipitation assay buffer
RT—Room temperature
SDS—Sodium dodecyl sulfate
SMA—Spinal muscular atrophy
SMN—Survival motor neuron
snRNA—small nuclear RNA
snRNP—Small U-type ribonucleoprotein
SSC—Saline-sodium citrate
STED—Stimulated emission depletion
TBST—Tween 20 and Tris-buffered saline
TVA—Transversus abdominis
WB—Western blot
WT—Wild-type
