# Supplementary material for: SMN Is Physiologically Downregulated at Wild-Type Motor Nerve Terminals but Aggregates Together with Neurofilaments in SMA Mouse Models
Source: Biomolecules. 2022 Oct 20;12(10):1524. doi: 10.3390/biom12101524 (PMC9599093; doi:10.3390/biom12101524)
Supplement: Supplementary file 1 [file biomolecules-12-01524-s001.zip › biomolecules-1941397-supplementary.pdf]

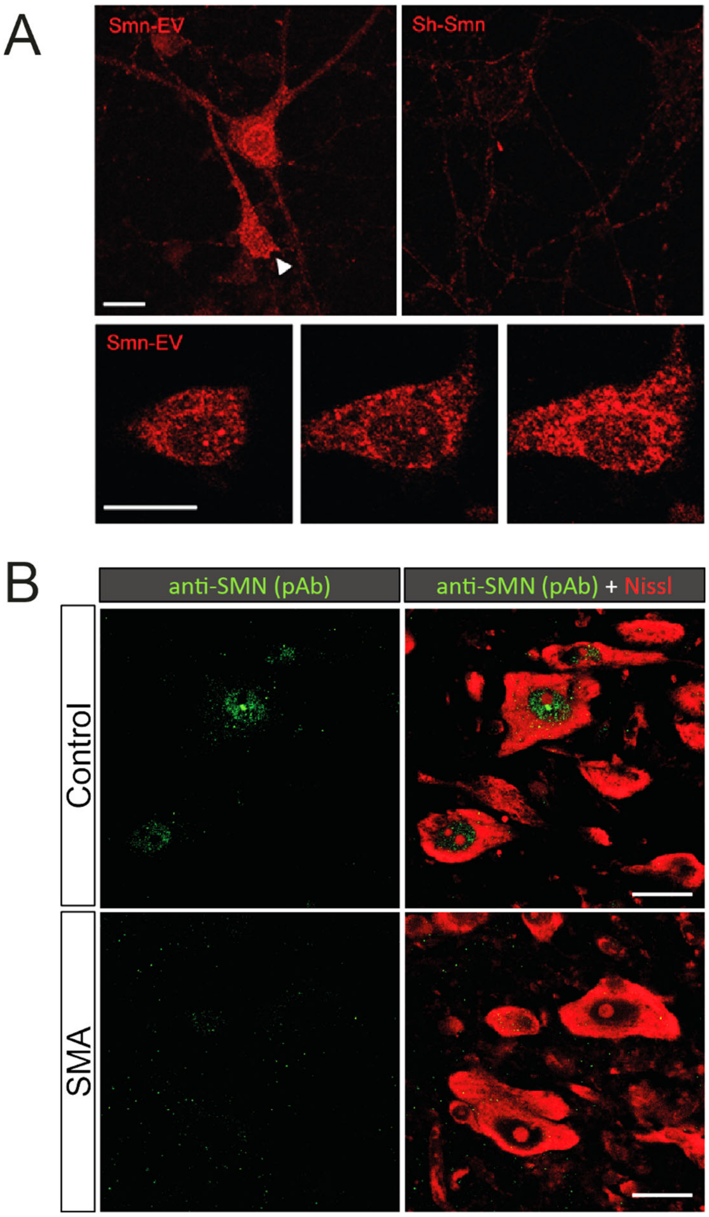

Figure S1. Validation of the anti-SMN H-195 polyclonal antibody (pAb). **A**. Confocal maximum intensity projection images of spinal motor neurons from embryonic wild-type mice in culture (DIV11). Upper panels: Motor neurons infected with an empty vector (EV) display signals at the soma, neurites, and growth cones (left, arrowhead). However, no such labeling is observed in Sh-Smn infected motor neurons (right). Lower panels: Examples of the Smn signal in the soma and within the nucleus (gems) at different planes of the same motor neuron. Scale bars: 20 µm. **B**. Confocal images of spinal cord sections from control and SMNΔ7 mice (P8) labeled with the anti-SMN pAb (green) and fluorescent Nissl staining (red) showing downregulation of the Smn signal in SMA motor neurons. Scale bars: 20 µm.

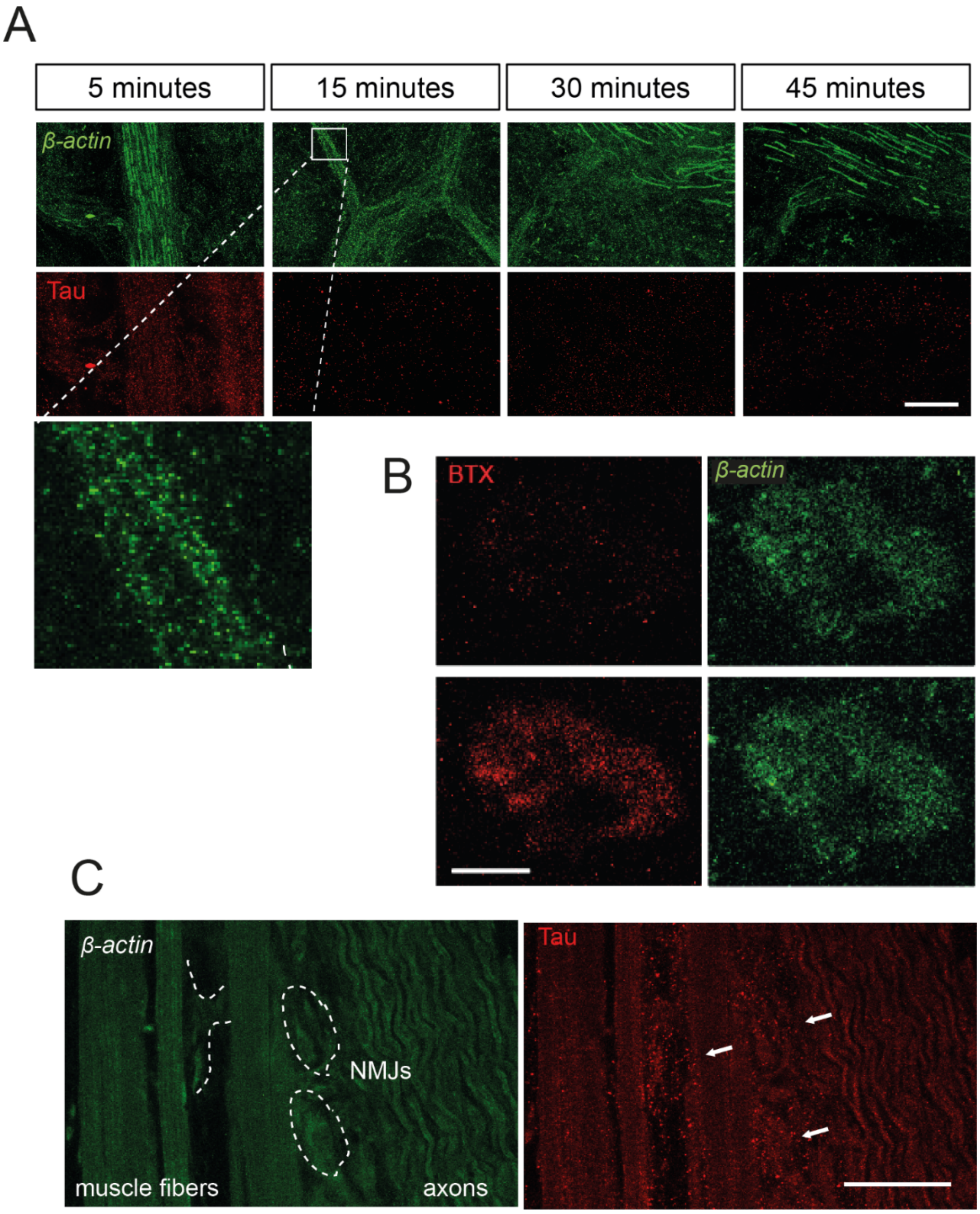

Figure S2. Establishment of experimental conditions for the detection of β-actin mRNA in neuromuscular preparations of TVA muscles. **A**. Treatment with 2 mg/ml of proteinase K for the indicated times. While the β-actin signal is evident at all times tested, only incubation with a short duration (5 min) allowed the visualization of the signal corresponding to Tau. The inset shows the dotted appearance of the β-actin mRNA signal. Scale bar: 40 µm. **B**. Bleedthrough and crosstalk between the red (BTX) and the green (β-actin) channels are negligible. If the tissue is excited exclusively at 488 nm, no signal is observed in the red channel (upper left panel). When 488 and 561 nm excitation was used, no appreciable change in the intensity of the β-actin signal was observed (lower right panel). Scale bars: 5 µm. **C**. Detection of the probe by the anti-DIG antibody is specific. Maximum intensity projections of a neuromuscular preparation are visualized in the green and red channels. The standard FISH protocol was applied but without the β-actin probe. Note that only green autofluorescence is visualized in the three NMJ in the field (outlined). At the same time, the Tau signal (red) corresponding to motor terminals at the NMJs are distinguished (arrows). Scale bar: 40 µm.

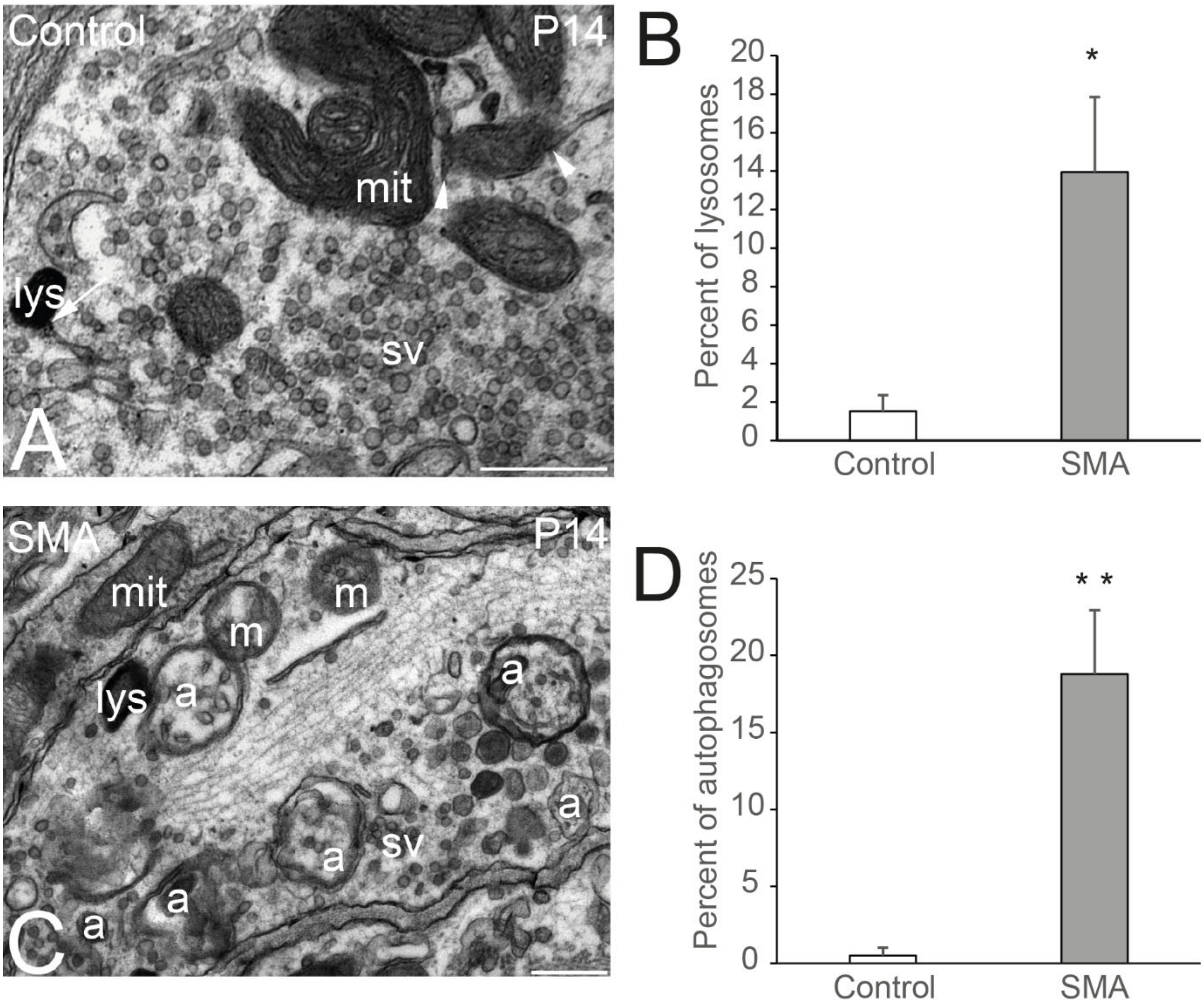

Figure S3. SMNΔ7 mutant and control presynaptic motor terminals contain lysosomes and autophagosomes. Electron microscopy representative images of NMJs from the TA muscle of control (**A**) and mutant SMNΔ7 (**C**) mice at P14. In the cytoplasm of the presynaptic terminal, synaptic vesicles (sv), mitochondria (mit), mitophagosomes (m), lysosomes (lys), and autophagosomes (a) were marked. Calibration bars: 500 nm. The graphs display quantification of lysosomes (**B**) and autophagosomes (**D**) in both genotypes (*: $p = 0.017$; **: $p = 0.004$).

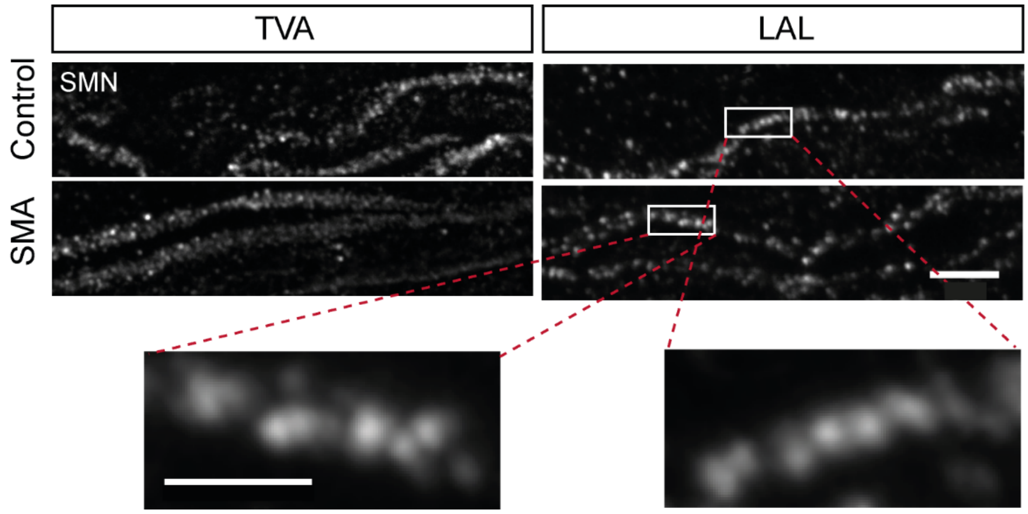

Figure S4. SMN granular pattern in the axons of control and SMA mice of two different muscles (TVA and LAL) at P9. The lowest panels show the delimited regions in greater detail. Scale bars: 5 μm (upper images) and 2 μm (lower images).

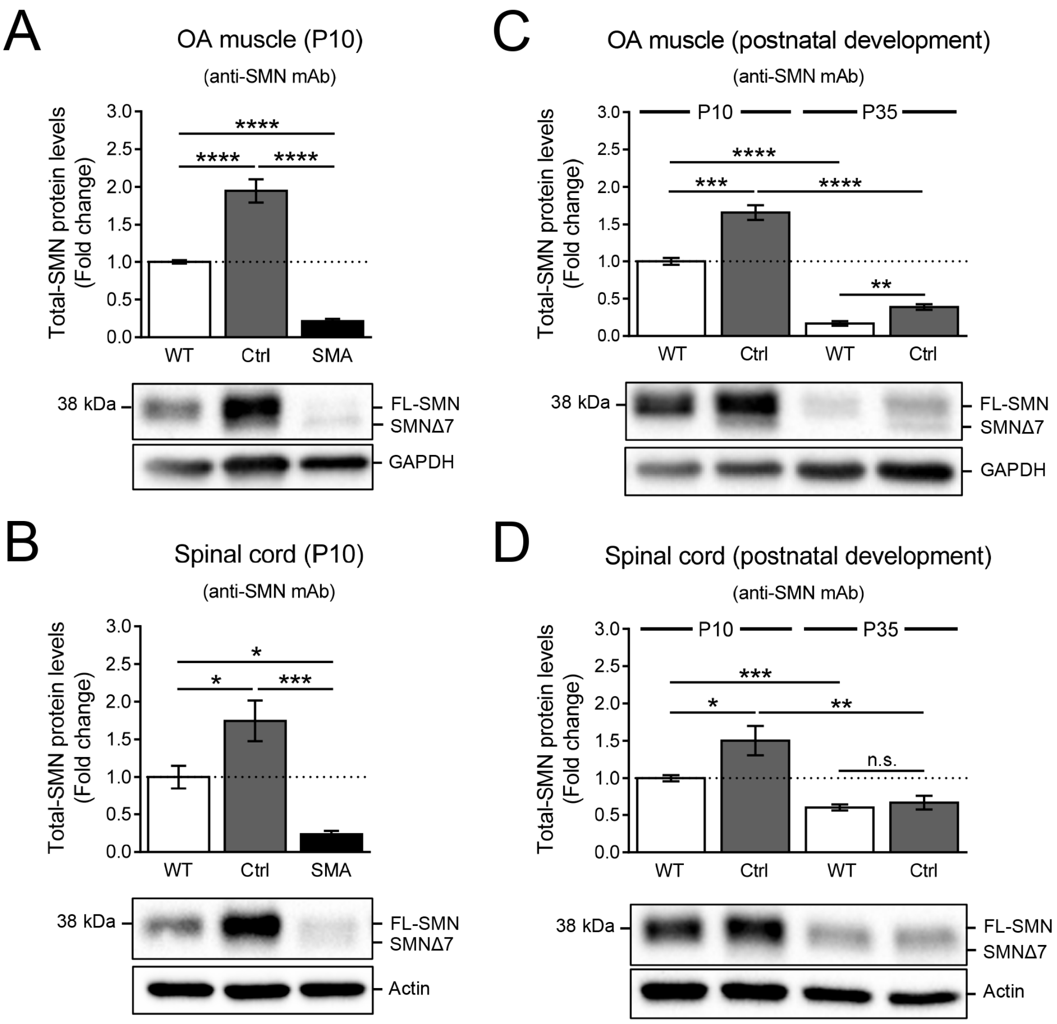

Figure S5. SMN protein levels in the OA muscle and the spinal cord of wild-type and transgenic SMNΔ7 mice. **A**–**B**. Quantification and representative images of western blot assays performed on obliquus abdominis (OA) muscle (**A**) and spinal cord (**B**) extracts obtained from P10 wild-type (WT) and transgenic SMNΔ7 mice (control [Ctrl] and SMA). A mouse monoclonal antibody (mAb) anti-SMN was used. Note that, in both, the OA muscle (**A**) and the spinal cord (**B**), total-SMN levels are significantly increased in Ctrl, compared to WT (****$p < 0.0001$ vs. WT [OA muscle]; *$p < 0.05$ vs. WT [spinal cord]). In contrast, relative expression of total SMN in these tissues is dramatically reduced in SMA mice (****$p < 0.0001$ vs. WT or Ctrl [OA muscle]; *$p < 0.05$ vs. WT and ***$p < 0.001$ vs. Ctrl [spinal cord]). In all the cases, total-SMN levels (FL-SMN and SMNΔ7, when present) were quantified in 4 WT, 4 Ctrl and 7 SMA mice and normalized first, to loading control (GAPDH for muscle; Actin for spinal cord) and then, to the average of the WTs. Data are presented as mean ± SEM and were analyzed by using a one-way ANOVA (Bonferroni's post-hoc test). **C**–**D**. Quantification and representative images of western blot assays performed on OA muscle (**C**) and spinal cord (**D**) extracts obtained from P10 and young adult (P35) WT and Ctrl transgenic SMNΔ7 mice. SMN protein levels in both tissues are significantly lower at P35 than at P10, in either WT (****$p < 0.0001$ vs. P10 WT [OA muscle]; ***$p<0.001$ vs. P10 WT [spinal cord]) or Ctrl (****$p < 0.0001$ vs. P10 Ctrl [OA muscle]; **$p<0.01$ vs. P10 WT [spinal cord]). However, SMN levels in P35 control animals remain significantly higher in P35 OA muscles (**$p < 0.01$ vs. P35 WT) but not, in the spinal cord (ns, $p > 0.05$ vs. P35 WT). In all cases, total-SMN levels (FL-SMN and SMNΔ7, when present) were quantified in 4 mice per experimental condition and normalized first, to loading control (GAPDH for OA muscle; Actin for spinal cord) and then, to the average of the P10 WTs. Data are presented as mean ± SEM. Each two conditions were compared by using a two-tailed Student's t-test.

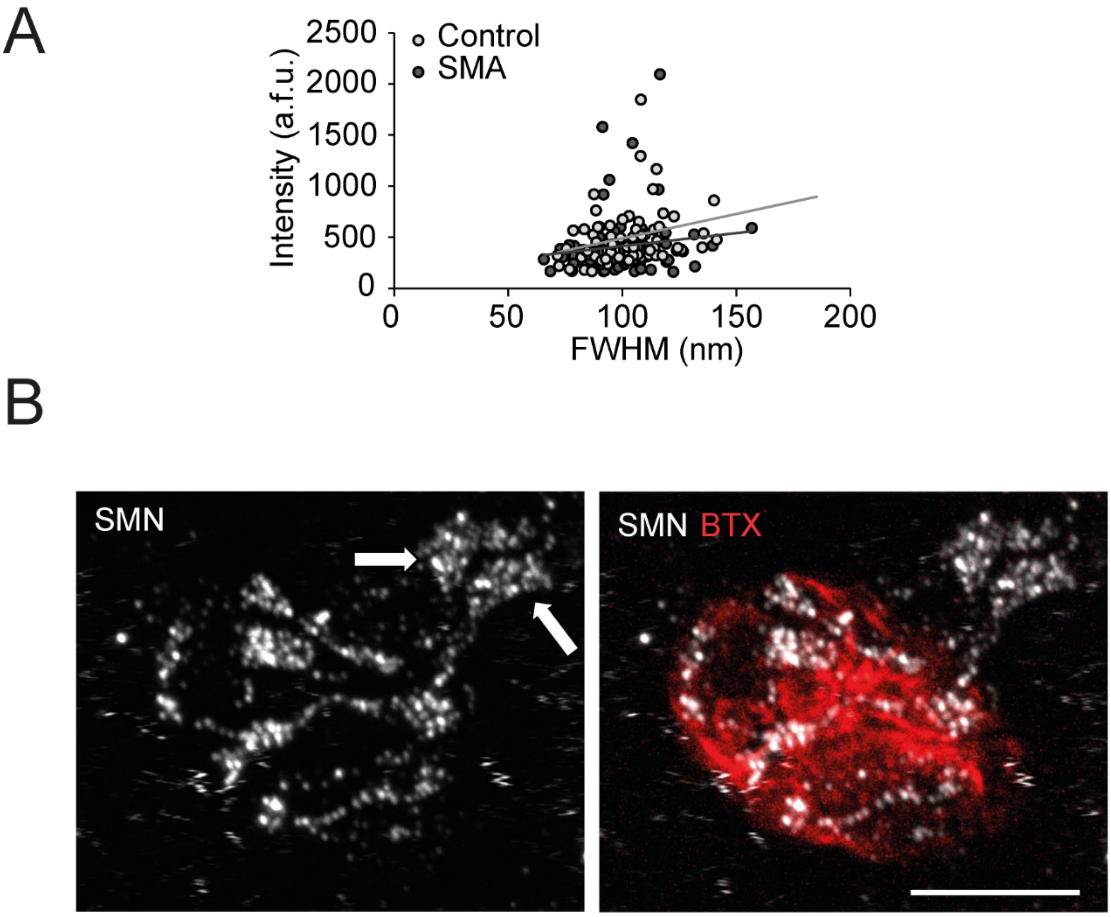

Figure S6. SMN size and cumuli. **A**. No correlation between the intensity and size of the SMN granules, measured as its FWHM, in either control and SMA mice **B.** Example of SMN accumulation at the distal portion of an axon (arrows). The right panel shows the merge of the presynaptic and the postsynaptic signals (BTX). Scale bar: 10 μm. a.f.u., arbitrary fluorescent units.
